# Parvovirus B19 infections in paediatric sickle cell disease patients: Genotype and hydroxyurea treatment influence disease severity

**DOI:** 10.1111/bjh.70809

**Published:** 2026-09-13

**Authors:** Matthias Bleeke, Arne Simon, Beate Winkler, Sabine Heine, Anna Eichinger, Ulrich von Both, Meinolf Siepermann, Sonya El Amouri, Arndt Borkhardt, Yvonne Pritschow, Wolfgang Novak, Michael Dworzak, Sarah Goretzki, Clara Held, Karin Beutel, Franziska Gams, Henriette Rudolph, Leila Koscher, Piet E. Beusker, Eckard Hamelmann, Stefan Weichert, Svenja Häuser, Maximilian Mauritz, Malik Aydin, Verena Wiegering, Regine Grosse, Rainer Misgeld, Martina Stiefel, Nicole Töpfner, Uta Falke, Lara Imhof, Laura Tagliaferri, Daniel Ebrahimi‐Fakhari, Andreas Groll, Marion Appel, Nora Naumann‐Bartsch, Michaela Nathrath, Olaf Beck, Solvej Heidtmann, Philipp Tischer, Ulf Schulze‐Sturm, Urs Mücke, Holger Cario

**Affiliations:** ^1^ Department of Pediatric Hematology and Oncology University Medical Center Hamburg‐Eppendorf Hamburg Germany; ^2^ Pediatric Hematology and Oncology Saarland University Hospital and Saarland University Faculty of Medicine Homburg Germany; ^3^ Department of Pediatrics, Dr. von Hauner Children's Hospital University Hospital LMU Munich Germany; ^4^ Department of Pediatric Hematology and Oncology Children's Hospital Amsterdamer Straße Cologne Germany; ^5^ Department of Pediatric Oncology, Hematology and Clinical Immunology, Medical Faculty Heinrich Heine University Düsseldorf Düsseldorf Germany; ^6^ Department of Pediatrics and Adolescent Medicine Ulm University Medical Center Ulm Germany; ^7^ Center for Rare Hematopoietic Disorders and Immunodeficiencies Ulm University Medical Center Ulm Germany; ^8^ Department of Pediatrics and Adolescent Medicine St. Anna Children's Hospital, Medical University of Vienna Vienna Austria; ^9^ Department of Pediatrics I, Neonatology, Pediatric Intensive Care, Pediatric Infectiology, Pediatric Neurology University of Duisburg‐Essen Essen Germany; ^10^ Department of Pediatrics, TUM School of Medicine and Health Technical University of Munich Munich Germany; ^11^ Department of Pediatrics Goethe‐University Frankfurt Frankfurt Germany; ^12^ Department of Pediatrics, Medical School and University Medical Center OWL, Protestant Hospital of the Bethel Foundation Bielefeld University Bielefeld Germany; ^13^ Department of Pediatric Oncology University of Mannheim Mannheim Germany; ^14^ Department of Pediatrics, Children's and Adolescent's Hospital Datteln Witten/Herdecke University Datteln Germany; ^15^ Department of Pediatric Hematology, Oncology and Stem Cell Transplantation, University Children's Hospital University of Würzburg Würzburg Germany; ^16^ Department of Pediatric Hematology and Oncology Gemeinschaftsklinikum Mittelrhein Koblenz Germany; ^17^ University Hospital Halle (Saale), Department of Pediatrics I Martin Luther University Halle‐Wittenberg Halle Germany; ^18^ Department of Pediatrics, Faculty of Medicine and University Hospital Carl Gustav Carus Technische Universität Dresden Dresden Germany; ^19^ Department of Pediatrics, University Hospital Aachen RWTH Aachen University Aachen Germany; ^20^ Department of Pediatric Oncology, Haematology and Immunology, Hopp‐Children's Cancer Center (KiTZ) Heidelberg University of Heidelberg Heidelberg Germany; ^21^ Pediatric Hematology and Oncology University Children's Hospital Muenster Muenster Germany; ^22^ Department of Pediatrics and Adolescent Medicine University Hospital Erlangen Erlangen Germany; ^23^ Department of Pediatric Hemato‐Oncology, Psychosomatics and Systemic Diseases Klinikum Kassel Kassel Germany; ^24^ Department of Pediatric Hematology/Oncology University Medical Center of the Johannes Gutenberg‐University Mainz Mainz Germany; ^25^ Department of Pediatrics Ruhr University Bochum, Johannes‐Wesling‐Klinikum Minden Minden Germany; ^26^ Department of Pediatrics University Medical Center Hamburg‐Eppendorf Hamburg Germany; ^27^ Department of Pediatric Hematology and Oncology Hannover Medical School Hannover Germany; ^28^ German Center for Child and Adolescent Health, Partner Site Ulm Ulm Germany

**Keywords:** parvovirus B19, sickle cell disease

## Abstract

In patients with sickle cell disease (SCD), parvovirus B19 infection (B19V) leads to acute anaemia (aplastic crisis), but may also be associated with other serious complications. We retrospectively analysed clinical data from paediatric SCD patients with B19V infections between 2023 and 2025, including symptoms, laboratory parameters, diagnostic procedures and treatment details to detect differences between SCD genotypes (HbSS and HbS/β^0^‐thalassaemia, i.e. HbSS/Sβ^0^, and HbSC) and to determine the effect of hydroxyurea (HU; hydroxycarbamide) treatment in HbSS/Sβ^0^ patients. Ninety‐eight patients (76 HbSS/Sβ^0^, 22 HbSC) were included in the study. There were significant differences in the symptoms of infection between genotypes, with a high prevalence of back pain as well as higher C‐reactive protein and lactate dehydrogenase levels in HbSC. HbSC patients also received chest X‐ray more frequently and required longer inpatient treatment. In addition, HbSC patients showed a significantly higher rate of immunoglobulin G seroconversion at the onset of symptoms, suggesting that antibody formation influences clinical appearance. In HbSS/Sβ^0^ patients, HU treatment was associated with higher haemoglobin and reduced transfusion requirements, but more frequently with neutropenia. In summary, B19V infections induce different clinical presentations depending on SCD genotypes, with HbSC patients showing a distinct phenotypic presentation. HU treatment reduces the need for transfusions in patients with HbSS/Sβ^0^.

## INTRODUCTION

Human parvovirus B19 (B19V) causes *erythema infectiosum*, also known as ‘fifth disease’. In otherwise healthy individuals, the infection is biphasic: a first phase characterized by non‐specific symptoms, such as general malaise and fever. As soon as specific antibodies are detectable, a typical rash or arthralgia may appear.^1^

The virus has a tropism for early erythropoietic progenitor cells, representing the exclusive site of its replication.^2^ Therefore, in patients with pre‐existing haemolytic anaemias such as sickle cell disease (SCD), the consecutive interruption of erythropoiesis for 7–10 days may lead to an acute worsening of anaemia (i.e. aplastic crisis).^3^, ^4^

SCD is caused either by a homozygous mutation in *HBB, which codes for beta‐globin chains* (c.20A>T or p.Glu7Val), or by compound‐heterozygosity for this mutation and a second *HBB* mutation, such as a β‐thalassaemia variant or another haemoglobin variant like haemoglobin C causing HbSC disease.^5^ In addition, the clinical course of SCD is further influenced by additional genetic, e.g. hereditary persistence of foetal haemoglobin (HPFH) or α‐thalassaemia, or environmental factors and especially treatment with hydroxyurea (HU; hydroxycarbamide).^6^, ^7^

Clinical complications of B19V infections in SCD patients include vaso‐occlusive events (VOE), acute chest syndrome (ACS), acute splenic sequestration and stroke. Interestingly, patients with HbSC disease have been reported to be susceptible to a more complicated course of infection than patients with HbSS genotype.^8^, ^9^, ^10^ However, systematic studies on B19V infections in SCD are scarce, especially studies comparing the clinical presentation across SCD genotypes.^11^, ^12^

B19V exhibits a consistent baseline infectious activity, with clusters of infections occurring every 4–5 years.^13^ It is a typical childhood infection with high seroconversion rates in early adulthood resulting in a lifelong immunity.^14^ Under usual conditions with sporadically occurring B19V infections, the collection of data on a representative cohort of acutely infected SCD patients requires a long period of time which may be associated with changing environmental or health‐care related conditions that might influence disease course and outcome. B19V infection rates decreased during the 2020–2022 SARS‐CoV‐2 pandemic, but after cessation of public protection measures against SARS‐CoV‐2, several countries reported a massive surge of B19V cases in 2023 and 2024 representing a unique opportunity to gather data on B19V infections in a defined period of time.^15^, ^16^, ^17^, ^18^

To gain an overview of the clinical presentation of B19V infections in a contemporary cohort of children and adolescents with SCD we invited paediatric haematology centres in Germany and Austria to a retrospective multicentre survey. This study aimed to describe the differences in the clinical presentation and course of B19V infections in patients with HbSS/Sβ^0^ and HbSC disease, and to investigate the influence of HU treatment.

## METHODS

### Data collection

A retrospective, cross‐sectional survey was conducted among 26 paediatric centres in Germany and Austria on clinical parameters of acute B19V infections in children and adolescents with SCD occurring between September 2023 and May 2025. Clinical data were collected using a REDCap database, hosted at University Medical Center Hamburg‐Eppendorf.^19^ Patients with SCD of different genotypes, aged between >28 days and 18 years, who had evidence of a recent B19V infection, as demonstrated by positive B19V‐specific serum immunoglobulin M (IgM) and/or B19V polymerase chain reaction (PCR), were included in the study. Data entry was performed retrospectively and anonymized by the participating centre and data were obtained from clinical care records. The following data were collected: age, gender, genotype, clinical symptoms at the time of infection detection, type and findings of virological analyses, laboratory parameters at the time of infection detection and during the course of the infection as well as information on further diagnostics and treatment.

The study was approved by the ethics committee or institutional review board at each participating centre. The study is registered in the German Clinical Trials Registry (DRKS00036034).

### Statistical analyses

Before analysing the data, the datasets were analysed for consistency and the units of measurement for laboratory values were converted to conventional units. Viral load data exceeding the centre's range of measurement were adjusted to the corresponding upper limit of detection. Unless otherwise specified, one red blood cell concentrate was assumed to equal 300 mL when calculating cumulative transfusion volumes. Patients with genotypes HbSS and HbS‐beta^0^ thalassaemia were categorized as ‘HbSS/Sβ^0^’ given their largely identical clinical SCD phenotype. Statistical analyses were performed using SPSS 29.0 (IBM Corp., Armonk, NY, USA). Nominal and categorical variables were compared using the chi‐square test or Fisher's exact test. Metric variables were analysed using non‐parametric tests (Mann–Whitney or Kruskal–Wallis tests). A *p*‐value <0.05 was considered statistically significant.

## RESULTS

### Patient population

A total of 100 patients with SCD were included in the study. Two patients with sole IgM titres without clinical evidence of an acute infection were excluded from the analysis. Of the 98 remaining patients, 75 had an HbSS and one had an HbS‐beta^0^ genotype and 22 patients presented with HbSC disease. Gender distribution was balanced in HbSS/Sβ^0^ with 48.7% female patients, whereas there was a slight predominance of male patients in HbSC. The median age at B19V infection was 7.3 and 10.3 years for HbSS/Sβ^0^ and HbSC patients, respectively (*p* < 0.01). Prior to the B19V infection, the diagnosis of SCD was unknown in 12 patients of the HbSS/Sβ^0^ group and in six patients of the HbSC group (15.8% and 27.3%, respectively). At the time of infection, 74.2% of HbSS/Sβ^0^ patients and 22.2% of HbSC patients were receiving HU treatment. The median HU dose in treated patients in these groups was 20.9 mg/kg/day and 18.8 mg/kg/day, respectively (Table 1).

**TABLE 1:** Demographics of patients with HbSS/Sβ^0^ and HbSC genotype.

Genotype	HbSS/Sβ^0^, *n* = 76	HbSC, *n* = 22	*p*
Gender: female	37 (48.7%)	7 (31.8%)	0.224
Age at SCD diagnosis [years]	2.0 (0.7; 3.2)	5.8 (1.2; 9.2)	**0.007**
Age at B19V infection [years]	7.3 (5.2; 9.3)	10.3 (7.2; 13.9)	**0.003**
HU treatment	49/66 (74.2%)	4/18 (22.2%)	**< 0.001**
HU Dose [mg/kg/day]	20.9 (16.6; 27.1)	18.8 (16.2; 23.6)	0.444
Previous HSCT	2 (2.6%)	0	1.000
Previous splenectomy	3/75 (4.0%)	0	1.000
SCD not known before infection	12 (15.8%)	6 (27.3%)	0.226

*Note*: All metric parameters are expressed as medians with first and third quartiles in parentheses. Data on HU treatment was available for 84 patients (including patients with previously undiagnosed SCD). Both patients with previous HSCT experienced secondary graft failure and are therefore considered as having HbSS phenotype. Statistically significant differences between groups are (*p* < 0.05) are shown in bold type.

Abbreviations: *B19V*, human parvovirus B19; HSCT, haematompoietic stem cell transplantation; HU, hydroxyurea (hydroxycarbamide); *n.a*., not applicable; *SCD*, sickle cell disease.

### Clinical presentation of B19V infection

The most frequently reported symptoms of B19V infections across all genotypes were pain (68.8%), fever (54.1%) and fatigue (36.7%). When considered separately according to genotype, fever and pain were the most commonly reported symptoms in HbSS/Sβ^0^ patients with 60.5% and 57.9% respectively, followed by fatigue (42.1%). In patients with HbSC disease, pain was by far the most frequently reported symptom (95.5%), followed by fever (31.8%) and fatigue (18.2%) (Figure 1A). There were marked differences in pain site distributions between the two groups of patients. In HbSS/Sβ^0^ patients, limb pain, abdominal pain and headaches were reported with similar frequencies of 47.7%, 38.6% and 38.6%, respectively. In contrast, HbSC patients reported back pain as the most common pain location with 71.4%. Overall, back and chest pain were significantly more prevalent in HbSC than in HbSS/Sβ^0^ patients, whereas headache was more frequent in HbSS/Sβ^0^ patients (Figure 1B).

**FIGURE 1:**
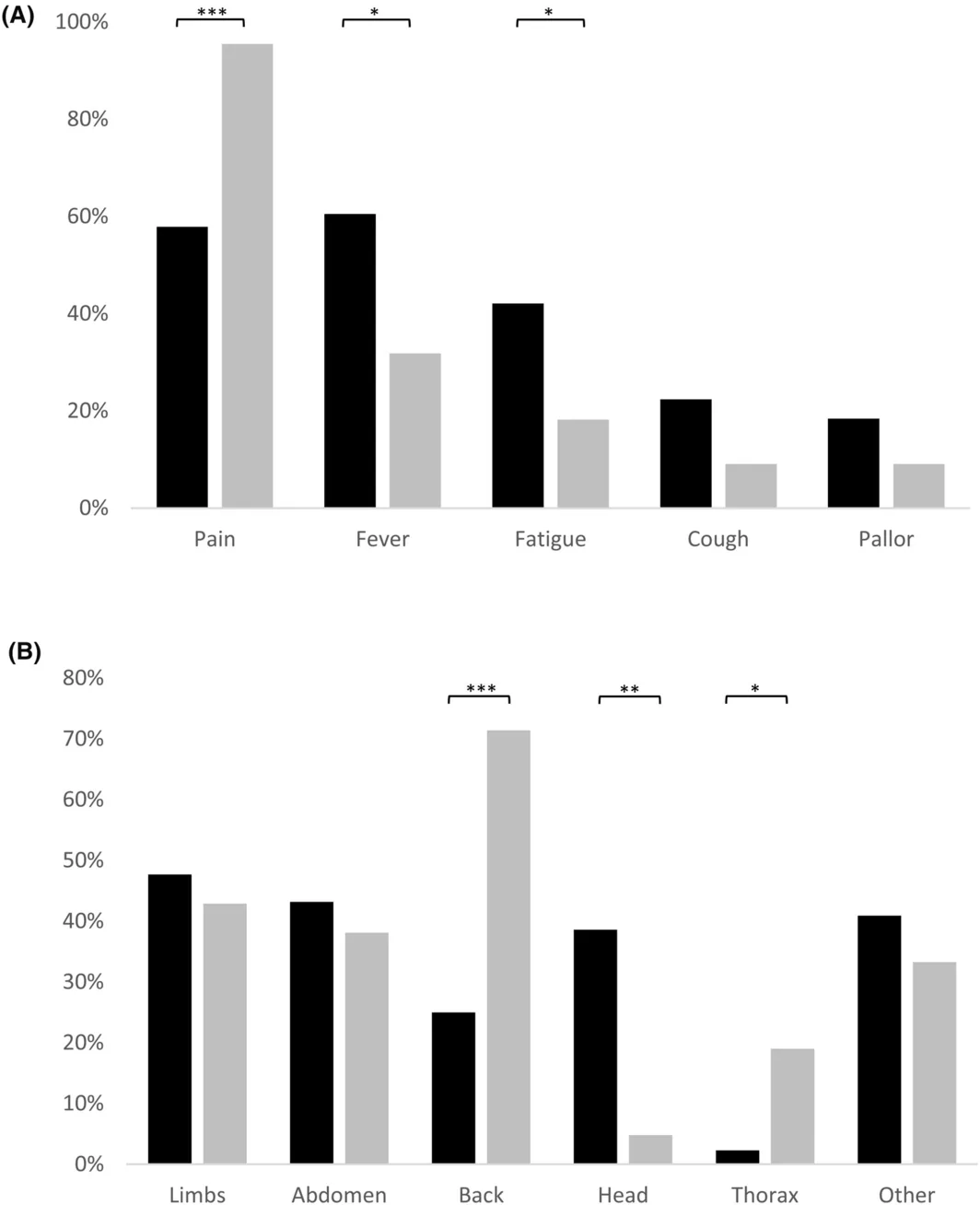
Symptoms at the onset of B19V infection in patients with HbSS/Sβ^0^ (black) and HbSC (grey) genotypes. The five most commonly reported symptoms of B19V infection for both genotypes are shown (panel A). Among 64 patients with pain among the reported initial symptoms, back and thorax were the most frequently reported pain locations in group 2 while group 1 patients showed a more heterogenous distribution of pain sites (panel B). *p < 0.05, **p < 0.01, ***p < 0.001.

### Virological diagnosis of B19V infection

B19V infection was detected serologically in 68.4% and by PCR in 81.6% of patients. The median interval between symptom onset and microbiological testing was 2 days (interquartile range [IQR] 1.0–4.5).

Considering patients with a maximum interval of 5 days between symptom onset and microbiological testing by serology and PCR, the serological B19V‐specific IgM and IgG pattern and viral loads were evaluated. Data from 36 patients (25 HbSS/Sβ^0^, 11 HbSC) met this criterion. HbSC patients were more likely to exhibit IgG seroconversion as compared to patients with HbSS/Sβ^0^ disease (Figure 2A).

**FIGURE 2:**
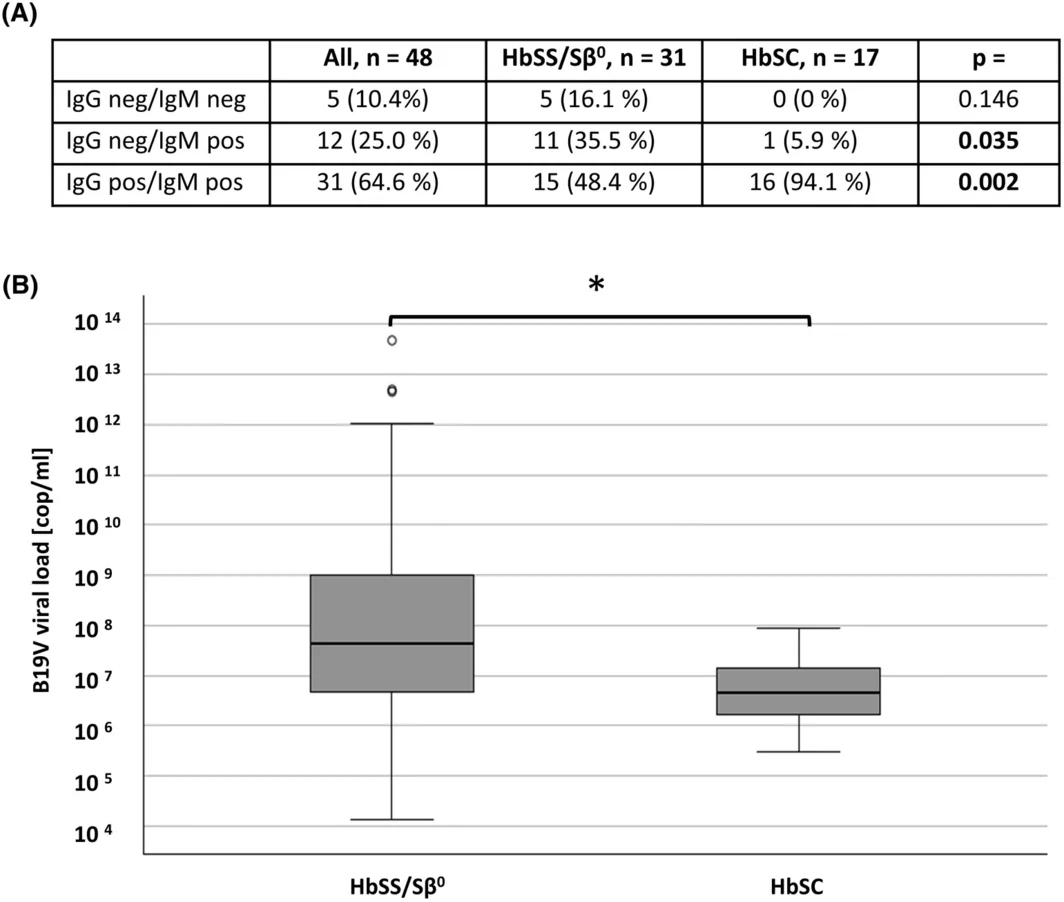
(A) Results of serological testing for 48 patients B19V‐specific antibodies in HbSS/Sβ^0^ and HbSC with a maximum interval between symptom onset and serological testing of 5 days. Serological patterns were grouped according to presence of B19V‐IgM, ‐IgG or both resembling earlier or later stages of the infection. (B) Viral loads as determined by PCR for 41 HbSS/Sβ^0^ patients and 11 HbSC patients with a maximum interval of 5 days between symptom onset and PCR testing. *p < 0.05.

The parallel analysis of viral loads revealed a higher median viral load in HbSS/Sβ^0^ patients as compared to HbSC patients (1.8 × 10^7^ cop/mL and 2.98 × 10^6^ cop/mL, respectively; *p* = 0.031) (Figure 2B).

### Blood counts and laboratory assessments

At the time of B19V diagnosis, patients with HbSS/Sβ^0^ disease showed significantly lower haemoglobin levels than patients with HbSC disease. Reticulocytopenia, as expected in aplastic crises, was observed in both groups. Haemoglobin nadir levels in HbSS/Sβ^0^ mostly corresponded to initial haemoglobin, while haemoglobin decreased further before nadirs were reached in HbSC disease. Neutrophil nadirs were significantly lower in HbSS/Sβ^0^ patients. Episodes of neutropenia and thrombocytopenia occurred rather frequently in both groups during the course of the B19V infection, with a higher incidence of neutropenia in HbSS/Sβ^0^ (Table 2).

**TABLE 2:** Blood counts at B19V infection diagnosis and occurrence of neutropenia and thrombocytopenia associated with B19V infections.

	All	HbSS/Sβ^0^	HbSC	*p*
Blood counts				
Haemoglobin, *n*	98	76	22	
Initial [g/dL]	6.1 (4.3; 7.9)	5.0 (3.8; 7.1)	8.0 (6.8; 9.0)	**< 0.001**
Nadir [g/dL]	5.8 (4.4; 6.5)	4.9 (3.7; 6.3)	6.4 (5.8; 7.0)	**< 0.001**
WBC, *n*	97	75	22	
Initial [/nL]	7.4 (4.8; 14.0)	7.0 (5.0; 14.7)	9.4 (4.8; 13.9)	0.563
Nadir [/nL]	5.1 (3.6; 7.4)	5.3 (3.6; 9.0)	6.2 (3.5; 8.8)	0.990
ANC, *n*	87	66	21	
Initial [/nL]	3.6 (2.2; 6.6)	3.3 (2.2; 5.7)	5.3 (2.8; 7.2)	0.167
Nadir [/nL]	2.2 (1.1; 3.6)	1.8 (0.9; 3.3)	3.1 (2.2; 4.1)	**0.023**
Thrombocytes, *n*	97	75	22	
Initial [/nL]	223 (124; 389)	220 (123; 371)	236 (152; 432)	0.433
Nadir [/nL]	157 (92; 276)	177 (99; 319)	163 (103; 214)	0.853
Reticulocytes, *n*	89	68	21	
Initial [%]	0.5 (0.2; 1.4)	0.5 (0.3; 1.4)	0.3 (0.2; 0.9)	0.228
Initial [/nL]	8.0 (4.0; 27.9)	7.8 (4.1; 23.3)	10.1 (6.3; 28.0)	0.432
Cytopenias associated with B19V infections				
ANC <1.5/nL	29 (33.3%)	26 (39.4%)	3 (14.3%)	**0.037**
ANC <1.0/nL	20 (23.0%)	19 (28.8%)	1 (4.8%)	**0.034**
ANC <0.5/nL	7 (8.0%)	6 (9.1%)	1 (4.8%)	1.000
Thrombocytes <150/nL	43 (44.3%)	35 (46.7%)	8 (36.4%)	0.469
Thrombocytes <100/nL	25 (25.8%)	20 (26.7%)	5 (22.7%)	0.789
Thrombocytes <50/nL	8 (8.2%)	7 (9.3%)	1 (4.5%)	0.678

*Note*: Initial and nadir haemoglobin, WBC, ANC, thrombocytes and reticulocytes are expressed as medians with first and third quartiles in parentheses. Relative reticulocyte counts in % are presented as reported by the participating centres. Absolute reticulocyte counts were calculated based on reported erythrocyte counts. Classification with regard to cytopenia was based on the reported nadirs of neutrophils and thrombocytes in each case and is expressed as absolute and relative incidences related to all patients with neutrophil or thrombocyte counts available. Statistically significant differences between groups are (*p* < 0.05) are shown in bold type.

Abbreviations: ANC, absolute neutrophil count; WBC, white blood cell count.

Maximum C‐reactive protein (CRP) was significantly higher in HbSC than HbSS/Sβ^0^ with median values of 70.1 mg/L and 13.0 mg/L respectively (*p* = 0.007). There was no indication for additional infections other than B19V as an underlying cause of higher CRP (Table S1). Significant differences were also observed in alanine aminotransferase (ALAT) although both groups' median levels remained within the normal range. Lactate dehydrogenase (LDH) was slightly but not significantly higher in HbSC than in HbSS/Sβ^0^ (850 vs. 659 U/L, *p* = 0.366) with nearly equal total bilirubin levels (1.4 vs. 1.2 mg/dL, *p* = 0.408) (Figure 3).

**FIGURE 3:**
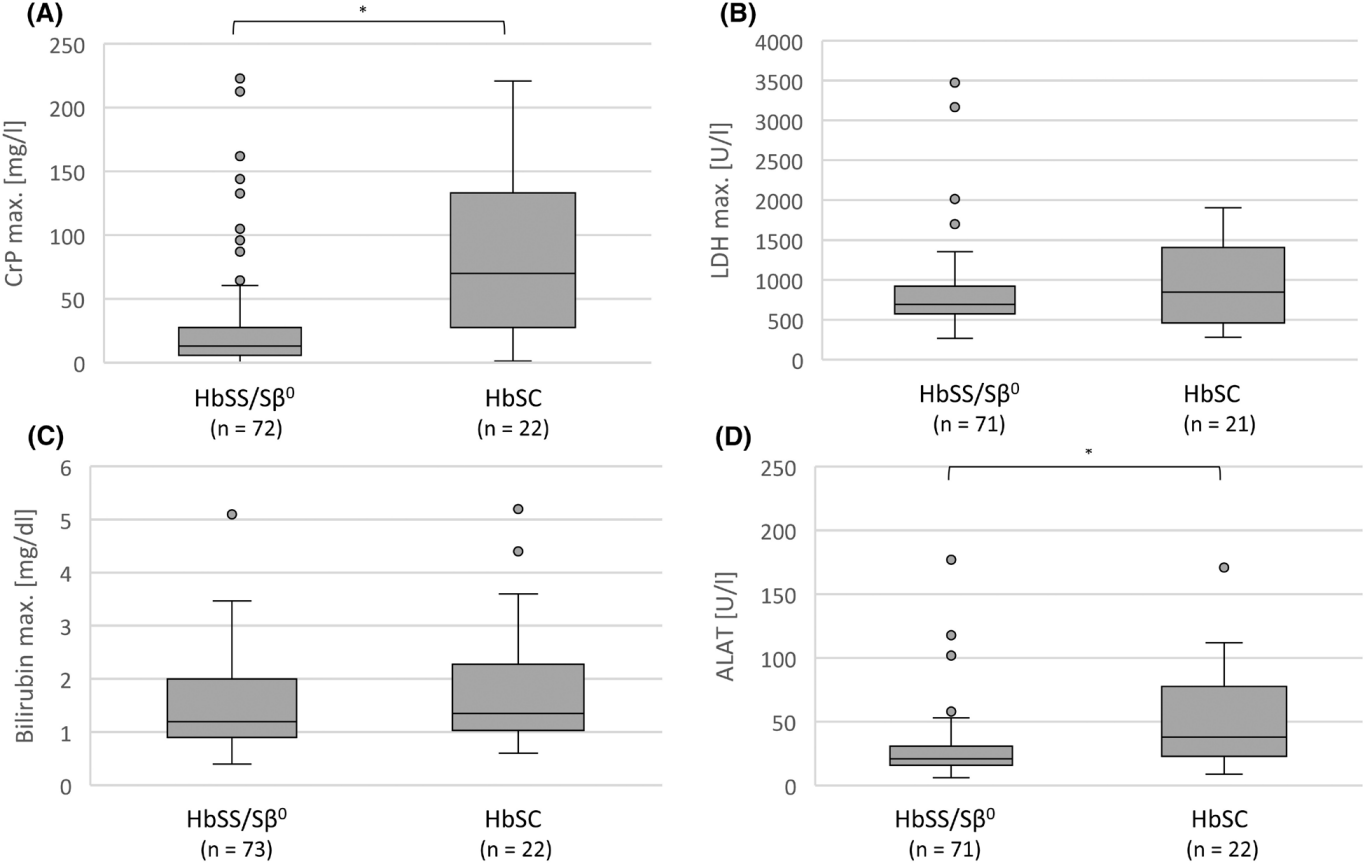
Further laboratory assessments in HbSS/Sβ^0^ and HbSC patients. Box plots represent C‐reactive protein (CRP, A), lactate dehydrogenase (LDH, B), total bilirubin (C) and alanine aminotransferase (ALAT, D). All parameters were recorded as maximum values during the course of the infection and converted to conventional units before analysis. *p < 0.05.

### Clinical course and complications

Blood transfusions were given to 85.5% of HbSS/Sβ^0^ patients including three emergency transfusions without prior cross‐matching, and to 68.2% of HbSC patients. The cumulative transfusion volume was 15.7 mL/kg in HbSS/Sβ^0^ and 16.4 mL/kg in HbSC (*p* = 0.270). One patient in the HbSS/Sβ^0^ group received red cell exchange transfusion.

Chest X‐rays were performed more often in HbSC patients (12/22 vs. 14/76, *p* = 0.002). Ten patients in the HbSC group, but only seven patients in the HbSS/Sβ^0^ group had radiographic evidence of pulmonary infiltrates. Based on the total group sizes, 45.5% in HbSC and 9.2% in HbSS/Sβ^0^ were diagnosed with pulmonary infiltrates (*p* < 0.001), while additional microbiological testing did not reveal a relevant incidence of additional respiratory infections (Table S1).

Inpatient treatment was required for 78.9% and 90.9% of patients in HbSS/Sβ^0^ and HbSC respectively (*p* = 0.348). Inpatient treatment was significantly longer in the HbSC group than in the HbSS/Sβ^0^ group with a median duration of 7 days (IQR 6–9.5) and 4 days (IQR 3–7) respectively (*p* < 0.001). Admission to paediatric intensive care occurred in 13.5% and 11.1% of cases in both groups, respectively (*p* = 1.000).

### Effect of HU treatment on B19V infections in HbSS/Sβ^0^ genotype

Information on HU treatment was available for 66 patients with HbSS/Sβ^0^ disease, with 49/66 patients receiving HU treatment at the time of B19V infection. In 12/17 HU‐naïve patients, the SCD was not yet diagnosed before the occurrence of B19V infection.

The median daily HU dose was 20.9 mg/kg. There were no significant differences regarding clinical symptoms of B19V infections at first presentation between patients with and without HU treatment (data not shown). Patients receiving HU treatment for HbSS/Sβ^0^ showed higher haemoglobin and lower total leucocyte counts in their initial blood tests than those not receiving HU treatment. There was no significant difference with regard to CrP, LDH or total bilirubin levels (Table 3).

**TABLE 3:** Clinical course of B19V infection in HbSS/Sβ^0^ patients depending on HU treatment.

	HbSS/Sβ^0^, HU +, *n* = 49	HbSS/Sβ^0^, HU –, *n* = 17	*p*
Patient characteristics			
Gender: female	19 (38.8%)	12 (70.6%)	**0.028**
Age at B19V infection [years]	7.4 (5.6; 9.3)	5.4 (3.1; 7.4)	**0.042**
HU Dose [mg/kg/day]	20.9 (17.7; 27.1)	n.a.	n.a.
SCD not known before infection	0	12 (70.6%)	<0.001
Blood count at initial presentation			
Haemoglobin [g/dL]	*n* = 47 5.1 (4.3; 7.6)	*n* = 17 4.7 (2.6; 5.8)	**0.013**
WBC [/nL]	*n* = 47 6.3 (4.2; 12.5)	*n* = 16 11.4 (7.2; 29.1)	**0.003**
ANC [/nL]	*n* = 41 3.0 (2.0; 5.7)	*n* = 15 4.5 (2.6; 12.3)	0.157
Thrombocytes [/nL]	*n* = 49 210 (123; 315)	*n* = 17 230 (178; 400)	0.315
Reticulocytes, relative [%]	*n* = 44 0.5 (0.3; 1.4)	*n* = 16 0.5 (0.3; 2.1)	0.763
Reticulocytes, absolute [/nL]	7.5 (3.7; 25.2)	6.4 (4.6; 28.3)	0.949
Laboratory parameters			
CrP, max. [mg/L]	*n* = 46 13.5 (7.0; 27.0)	*n* = 17 10.0 (4.0; 33.0)	0.828
LDH, max. [U/L]	*n* = 46 689 (567; 900)	*n* = 17 776 (630; 1030)	0.161
Total bilirubin, max. [mg/dL]	*n* = 47 1.1 (0.9; 2.0)	*n* = 17 1.3 (1.1; 1.7)	0.159
Treatment			
Hospitalization	36 (73.5%)	17 (100%)	**0.015**
Intensive care treatment	3 (9.7%)	4 (26.7%)	0.144
Duration of hospitalization	5.0 (3.0; 7.0)	4.0 (3.0; 6.0)	0.298
RBC transfusion	40 (81.6%)	16 (94.1%)	0.432
RBC transfusion volume [ml/kg]	15 (12.5; 21.7)	21.5 (15.7; 29.0)	**0.017**

*Note*: Parameters of the clinical course of B19V infection in HbSS/Sβ^0^ patients with HU treatment (HU +) and without HU treatment (HU –). Metric scaled parameters are expressed as median with first and third quartiles in parentheses. The blood counts correspond to those at diagnosis of PB19 infection. The other parameters correspond to the maximums reported during the course of B19V infection treatment. Since not all laboratory parameters were available for all patients in each group, the number of available values (*n*) is provided for each laboratory parameter. Statistically significant differences between groups are (*p* < 0.05) are shown in bold type.

Abbreviations: ANC, absolute neutrophil count; CRP, C‐reactive protein; HU, hydroxyurea (hydroxycarbamide); LDH, lactate dehydrogenase; n.a., not applicable; RBC, red blood cell; SCD, sickle cell disease; WBC, white blood cell count.

Neutrophil and thrombocyte counts were available for 57/66 and 65/66 HbS/Sβ^0^ patients respectively. Neutropenia occurred more frequently in patients treated with HU compared to HU naïve patients (*p* = 0.004) (Table 4). Thrombocytopenia occurred more frequently in patients under HU treatment, although the difference was not statistically significant (Table 4).

**TABLE 4:** Frequencies of neutropenia and thrombocytopenia.

Neutrophils	HbSS/Sβ^0^, HU +, *n* = 42	HbSS/Sβ^0^, HU –, *n* = 15	*p*
Neutropenia, all	21 (50.0%)	1 (6.7%)	**0.004**
ANC 1.0–<1.5/nL	4 (9.5%)	1 (6.7%)	1.000
ANC 0.5–<1.0/nL	12 (28.6%)	0 (0%)	**0.024**
ANC <0.5/nL	5 (11.9%)	0 (0%)	0.311

Thrombocytes	HbSS/Sβ^0^, HU +, *n* = 49	HbSS/Sβ^0^, HU –, *n* = 16	
Thrombocytopenia, all	25 (51.0%)	5 (29.4%)	0.162
PLT 100–<150/nL	10 (20.4%)	3 (18.8%)	1.000
PLT 50–<100/nL	9 (18.4%)	2 (12.5%)	0.718
PLT <50/nL	6 (12.2%)	0 (0%)	0.323

*Note*: Neutropenia and thrombocytopenia, subclassified as mild, moderate or severe, in patients with HbSS/Sβ^0^ genotypes who received HU before B19V infection (HU+) and patients without HU treatment (HU–). Comparisons for statistical significance of variations using Fisher's exact test. Statistically significant differences between groups are (*p* < 0.05) are shown in bold type.

Abbreviations: ANC, absolute neutrophil count; PLT, platelets.

Inpatient treatment was provided to 73.5% of patients treated with HU and in all patients not treated with HU (*p* = 0.015). The median duration of inpatient treatment was 5 days for HU‐treated patients and 4 days for HU‐naïve patients (*p* = 0.298). The majority of HbSS/Sβ^0^ patients received at least one blood transfusion: 81.6% of those receiving HU treatment and 94.1% of those not receiving HU treatment (*p* = 0.432). HU‐treated patients had a significantly lower median cumulative transfusion volume of 15.0 mL/kg than those without HU treatment, with a median cumulative transfusion volume of 21.5 mL/kg (*p* = 0.017) (Table 3).

## DISCUSSION

The surge of B19V infections in the years 2023/2024 presented a unique opportunity to investigate the impact of the infection in individuals with congenital blood disorders. To our knowledge, this is the most comprehensive collection of clinical data on B19V infections in paediatric SCD patients, and the first study to directly compare the clinical course of the infection in children and adolescents with HbSS/Sβ^0^ and HbSC genotypes in a contemporary patient cohort.

Interestingly, a significant proportion of patients were not diagnosed with SCD before the B19V infection, particularly of those with HbSC disease (Table 1). Newborn screening for SCD was introduced in Germany in 2021,^20^ but children born before its introduction or who immigrated after their birth were not systematically screened. Our data therefore show that even in countries with general SCD newborn screening, continuous ongoing diagnostic awareness is necessary to identify children affected by SCD, ideally before the onset of complications.

Pain, fever and fatigue were the most common symptoms associated with B19V infection, but with marked differences between SCD genotypes (Figure 1A). In HbSS/Sβ^0^, fever, fatigue and headache could be attributed to the acute infection and severe anaemia. Conversely, not only the high incidence of pain, but particularly the dominance of back pain in HbSC is noteworthy (Figure 1B). Therefore, the clinical presentation of B19V infection in paediatric SCD appears to depend on the SCD genotype. In addition, the duration of hospitalization was significantly longer in HbSC patients compared to HbSS/Sβ^0^, indicating a more complicated course of the disease.

The stage of infection at which patients presented for clinical care may be significant in interpreting the clinical differences of B19V infections between SCD genotypes. The relatively low rate of IgG seroconversions and the high viral load in HbSS/Sβ^0^ patients indicate an early stage in the course of the infection. In contrast, with IgG seroconversion and advanced viral clearance, these parameters suggest a later stage of infection in HbSC patients. Since typical findings of B19V infection in otherwise healthy individuals, such as the pathognomonic rash, are associated with the appearance of IgG antibodies against B19V^3^ it therefore seems possible that IgG antibodies influence the specific symptoms observed in HbSC.

In our cohort, HbSC patients showed higher CrP levels compared to HbSS/Sβ^0^ as well as LDH levels which were unusually high for HbSC disease as this genotype is associated with less haemolysis than HbSS.^21^ The low bilirubin levels in both groups support that LDH increase is not caused by haemolysis, but can probably be explained by tissue damage. Patients with non‐HbSS SCD and B19V infection have been reported to be at higher risk for bone marrow necroses (BMN) compared to HbSS patients^12^ and in this context, the high prevalence of back pain and elevated markers of tissue damage suggest the presence of BMN in our HbSC cohort. B19V associated BMN has been described primarily in adult HbSC patients, but our observations suggest that BMN might be more common in paediatric HbSC patients with symptomatic B19V infection than previously assumed. However, it must be noted that BMN can be definitely confirmed only by bone marrow trephine biopsy, which was not performed in any of the patients reported here.

The pathophysiology of BMN in HbSC patients with B19V infections remains unclear. Blood viscosity, direct cytopathic effects of B19V on endothelial cells and inflammatory activity in the course of antiviral defence might influence bone marrow perfusion and lead to ischaemia.^22^ The preserved spleen function in patients with HbSC disease and the associated ability to mount an antibody response has been discussed as a reason for intergenotype variability.^23^ The high prevalence of B19V specific IgG seroconversions in HbSC patients at clinical presentation in our cohort seems to support the role of antibody formation in this regard.

ACS and fat embolism syndrome (FES) are serious and potentially life‐threatening complications of BMN, both of which have been reported in association with B19V infections in sickle cell patients, in particular adults with HbSC disease.^9^, ^12^ Although we did not specifically collect data on respiratory function, the higher incidence of chest pain, the more frequent utilization of chest X‐rays in HbSC patients and the finding that 45% of HbSC patients had radiographic evidence of pulmonary infiltrates indicate respiratory compromise in these patients. Therefore, it is important to be aware of these complications also in children and adolescents with HbSC disease and B19V infection.

HU is the standard treatment for patients with SCD, reducing sickling and haemolysis. This, in turn, might increase resilience to B19V‐associated aplastic crisis in HbSS/Sβ^0^ patients under HU treatment. We therefore examined the effect of HU treatment on the clinical course of B19V infection in HbSS/Sβ^0^ patients. Regarding the initial symptoms of the infection, we did not observe differences in frequencies of the reported symptoms, including pain. However, patients under HU treatment presented with higher haemoglobin and lower transfusion requirement compared to patients without HU treatment. This is in line with findings of a previous study on B19V infection and HU treatment in HbSS patients.^24^

Compared to previous studies, we observed a higher incidence of neutropenia and thrombocytopenia,^9^ which might be attributed to the higher utilization of HU in patients of our present study. Although B19V exclusively replicates in erythroid progenitors, neutropenia and thrombocytopenia may occur in the course of the infection as a result of a direct or indirect cytopathological effect of B19V on other precursors in the bone marrow.^25^, ^26^, ^27^ While the higher haemoglobin and lower transfusion volumes could be attributed to reduced haemolysis, neutropenia is probably the result of additive myelotoxic effects of the B19V infection and HU treatment.

Our study has limitations, primarily due to the retrospective data collection and limitation to data from regular clinical care. Asymptomatic infections could not be systematically assessed by our study design and would require longitudinal observation studies, for example, with regard to a protective effect of HU treatment. Furthermore, no data on steady‐state haemoglobin or haemoglobin F values were collected for the patients studied here, which would have been helpful in the assessment of the effect of HU treatment.

In summary, our analysis showed that B19V infection in paediatric SCD patients causes a specific clinical phenotype, depending on the underlying SCD genotype. Patients with HbSC disease tend to have a more severe clinical course beyond the typical symptoms of a B19V‐associated aplastic crisis, which might be attributed to the occurrence of BMN. Regarding the effect of HU treatment on B19V‐associated aplastic crises in HbSS/Sβ^0^, we observed a positive effect of HU on haemolysis and transfusion requirements, but a higher incidence of neutropenia.

## AUTHOR CONTRIBUTIONS

**Arne Simon:** Investigation; conceptualization; writing – original draft. **Matthias Bleeke:** Conceptualization; methodology; formal analysis; project administration; writing – original draft; investigation; visualization. **Sabine Heine:** Conceptualization; investigation; writing – original draft. **Anna Eichinger:** Investigation. **Beate Winkler:** Conceptualization; writing – original draft; investigation. **Meinolf Siepermann:** Investigation. **Sarah Goretzki:** Investigation. **Wolfgang Novak:** Investigation. **Ulrich von Both:** Investigation. **Yvonne Pritschow:** Investigation. **Michael Dworzak:** Investigation. **Sonya El Amouri:** Investigation. **Clara Held:** Investigation. **Verena Wiegering:** Investigation. **Arndt Borkhardt:** Investigation. **Stefan Weichert:** Investigation. **Svenja Häuser:** Investigation. **Eckard Hamelmann:** Investigation. **Martina Stiefel:** Investigation. **Nicole Töpfner:** Investigation. **Laura Tagliaferri:** Investigation. **Regine Grosse:** Investigation. **Michaela Nathrath:** Investigation. **Rainer Misgeld:** Investigation. **Karin Beutel:** Investigation. **Daniel Ebrahimi‐Fakhari:** Investigation. **Leila Koscher:** Investigation. **Piet E. Beusker:** Investigation. **Olaf Beck:** Investigation. **Franziska Gams:** Investigation. **Uta Falke:** Investigation. **Andreas Groll:** Investigation. **Solvej Heidtmann:** Investigation. **Lara Imhof:** Investigation. **Maximilian Mauritz:** Investigation. **Nora Naumann‐Bartsch:** Investigation. **Holger Cario:** Investigation; conceptualization; formal analysis; writing – original draft. **Philipp Tischer:** Investigation. **Ulf Schulze‐Sturm:** Investigation. **Marion Appel:** Investigation. **Henriette Rudolph:** Investigation. **Urs Mücke:** Investigation. **Malik Aydin:** Investigation.

## CONFLICT OF INTEREST STATEMENT

All authors declare no conflict of interest.

## ETHICS STATEMENT

The study was approved by the ethics committee or institutional review board at each participating centre. The study is registered in the German Clinical Trials Register (DRKS00036034).

## Supporting information


**Table S1.** Additional microbiological testing and results in HbSS/Sβ^0^ and HbSC patients.

## Data Availability

Data are available from the corresponding author upon reasonable request.
