# Supplementary material for: Parvovirus B19 infections in paediatric sickle cell disease patients: Genotype and hydroxyurea treatment influence disease severity
Source: Br J Haematol. 2026 Sep 13;209(4):1845–55. doi: 10.1111/bjh.70809 (PMC13674147; doi:10.1111/bjh.70809)
Supplement: Supplementary file 1 [file BJH-209-1845-s001.docx]

**Supplementary Table:** Additional microbiological testing and results in HbSS/Sβ^0^ and HbSC patients.

|  | | **HbSS/Sβ^0^** , n = 76 | | | **HbSC**, n = 22 | |
| --- | --- | --- | --- | --- | --- | --- |
| **Procedure** | | **Performed** | | **Positive** | **Performed** | **Positive result** |
| **Blood culture** | | 27 (35.5 %) | | 2 (2.6 %) | 13 (59.1 %) | 0 (0 %) |
|  | Pathogens detected |  | | M. luteus  S. haemolyticus |  |  |
| **Urine culture** | | 3 (3.9 %) | | 0 | 1 (4.5 %) | 0 (0 %) |
| **Throat swab** | | 27 (35.5 %) | | 8 (10.5 %) | 11 (50.0 %) | 1 (4.6 %) |
|  | Pathogens detected |  | | Adenovirus (1)  Rhinovirus (1)  RSV (1)  hMPV (2)  M. pneumoniae (2)  S. pneumoniae (1) |  | Parainfluenzavirus 3 (1) |
| **Stool analysis** | | 1 (1.3 %) | | 0 | 1 (4.5 %) | 0 |
| **Serology** | | 19 (25.0 %) | | 5 (6.6 %) | 4 (18.2 %) | 0 |
|  | IgM detected |  | | CMV (3)  EBV (1)  HHV6 (2) |  |  |
| **PCR** | | 10 (13.2 %) | | 2 (2.6 %) | 3 (13.6 %) | 0 |
|  | Viruses detected |  | HHV6 (1)  EBV (1) | |  |  |

All reported testing was indicated clinically by the treating centre as part of patient treatment. The total number of tests performed is indicated in the left column in each group, the number of positive results is shown in the right column, respectively. Percentages in brackets refer to the total patient number of the respective group. The pathogens found are listed individually with the respective number of pathogen detections in brackets. *CMV* cytomegalovirus; *EBV* epstein-barr-virus; *HHV6* human herpes virus 6; *hMPV* human metapneumovirus; *RSV* respiratory syncytial virus.
